# Web 2.0 systems supporting childhood chronic disease management: A pattern language representation of a general architecture

**DOI:** 10.1186/1472-6947-8-54

**Published:** 2008-11-28

**Authors:** Toomas Timpka, Henrik Eriksson, Johnny Ludvigsson, Joakim Ekberg, Sam Nordfeldt, Lena Hanberger

**Affiliations:** 1 Department of Medical and Health Sciences, Linköping University, Linköping, Sweden; 2 Department of Computer and Information Science, Linköping University, Linköping, Sweden; 3 Department of Clinical and Experimental Medicine, Linköping University, Linköping, Sweden

## Abstract

**Background:**

Chronic disease management is a global health concern. By the time they reach adolescence, 10–15% of all children live with a chronic disease. The role of educational interventions in facilitating adaptation to chronic disease is receiving growing recognition, and current care policies advocate greater involvement of patients in self-care. Web 2.0 is an umbrella term for new collaborative Internet services characterized by user participation in developing and managing content. Key elements include Really Simple Syndication (RSS) to rapidly disseminate awareness of new information; weblogs (blogs) to describe new trends, wikis to share knowledge, and podcasts to make information available on personal media players. This study addresses the potential to develop Web 2.0 services for young persons with a chronic disease. It is acknowledged that the management of childhood chronic disease is based on interplay between initiatives and resources on the part of patients, relatives, and health care professionals, and where the balance shifts over time to the patients and their families.

**Methods:**

Participatory action research was used to stepwise define a design specification in the form of a pattern language. Support for children diagnosed with diabetes Type 1 was used as the example area. Each individual design pattern was determined graphically using card sorting methods, and textually in the form *Title, Context, Problem, Solution, Examples and References*. *Application references *were included at the lowest level in the graphical overview in the pattern language but not specified in detail in the textual descriptions.

**Results:**

The design patterns are divided into functional and non-functional design elements, and formulated at the levels of organizational, system, and application design. The design elements specify access to materials for development of the competences needed for chronic disease management in specific community settings, endorsement of self-learning through online peer-to-peer communication, and systematic accreditation and evaluation of materials and processes.

**Conclusion:**

The use of design patterns allows representing the core design elements of a Web 2.0 system upon which an 'ecological' development of content respecting these constraints can be built. Future research should include evaluations of Web 2.0 systems implemented according to the architecture in practice settings.

## Introduction

Chronic disease management is a global health concern. By the time they reach adolescence, 10–15% of all children live with a chronic disease [1]. Young people with chronic conditions are doubly disadvantaged – engaging in risky behaviours to at least similar rates as healthy peers, while having the potential for greater adverse health outcomes from these behaviours. The main focus in chronic disease management has been on medical care, with less attention being paid to psychosocial aspects of life with a chronic disorder. Nevertheless, the role of educational interventions in facilitating adaptation to chronic disease is receiving growing recognition, and current care policies advocate greater involvement of patients in self-care. There is also growing scientific evidence for the effectiveness of behavioural interventions including a wide range of factors central to coping with a chronic disease, e.g. self-efficacy, self-management of disease, family support, psychosocial well-being, reduced isolation, and social competence [2]. But in order to improve the life quality of children with a chronic disease in practice, better support of young people's emerging capacity for self-care is needed, which, in turn, requires improved means to facilitate the social and emotional coping of young people with a chronic disease. It is therefore necessary to focus attention on the interaction between adolescents with chronic conditions and the health systems, in a broad definition, that support them.

Information technology has undergone rapid development in recent decades, which has had significant impact on social life [3]. Technical advances have also provided a foundation for ubiquitous and proactive health systems that use data from multiple sources to supply individuals and communities with support to improve their state of health and avoid health risks [4]. These networked systems are characterized by being profusely connected to the world around them [5]. Web 2.0 is an umbrella term for new collaborative Internet services utilizing this set of technical progresses. The primary difference from the first generation of Internet applications is the increased user participation in developing and managing content, which changes the nature and value of the information [6,7]. Key elements of Web 2.0 include Really Simple Syndication (RSS) to rapidly disseminate awareness of new information; weblogs (blogs) to describe new trends, wikis to share knowledge, and podcasts to make information available on personal media players. In the context of education, students are not regarded as just recipients of education but are involved in collaboration in learning activities, expressed as eLearning 2.0 or Education 2.0 . The present study addresses the potential to develop Web 2.0 services for young persons with a chronic disease. It is acknowledged that the management of chronic disease is based on interplay between initiatives and resources on the part of patients, relatives, and health care professionals, where the balance in shared responsibilities shifts over time to the patients and their families. The aim of the study is to specify a general architecture for Web 2.0 systems supporting chronically ill children and their families. The architecture is to be compatible with the eHealth resolution (WHA58.28), approved by the World Health Assembly (WHA). This resolution is the background ministerial commitment of the United Nations/WHO in the eHealth area. According to the resolution , long-term strategic plans for developing services in the area of eHealth need to be formulated for all health sectors, and multisectorial collaborations with a view to improving compatibility of organizational, technical, and ethical guidelines need to be promoted. Other areas emphasised in the resolution are equitable access to eHealth services, in particular by vulnerable groups, and IT security. A key factor for the realization of these visions in implemented software is the availability of a general architecture outlining the system, its main functions related to service delivery, and its boundaries.

Data for the analyses were collected from the development of a Web 2.0 system for support of children with diabetes Type 1 in a defined Swedish community. Diabetes Type 1 is probably the most salient example of a chronic disease that requires daily medication and lifestyle management. Modern treatment of diabetes requires coherence with tailored insulin regimens including several daily insulin injections adapted to regular meals with certain content, daily self-care with repeated blood glucose measurements, and continuous learning about the treatment. There is increasing evidence that appropriate intensive treatment reduces long-term complications [8,9]. The Web 2.0 system was to support the formation of partnerships between children and adults having an interest in diabetes management in a specific community, with the clinical professionals providing the local clinical care, and expert knowledge. In parallel, through co-operation with the other diabetes care programs, the Web 2.0 system is to mediate alliances between individuals in different geographically defined communities in efforts to develop novel supportive environments for the care of chronic paediatric disease.

## Methods

Structured graphical languages are commonly used by information systems developers, especially in complex systems settings [10]. The Web 2.0 system was modelled using the pattern language approach. Pattern languages provide conceptual means for representation of composite architectures [11]. A common language for the representation of inter-connected design patterns makes it possible to communicate an overview of a Web 2.0 system and its functionality in a way that is understandable to lay stakeholders and design groups with different professional competences represented. Each pattern of the pattern language addresses a specific design problem and describes a possible solution.

### Data collection and analysis

Participatory action research (PAR) methods were used for the integrated development of the architecture, research data collection and analysis [12]. PAR is based on collaboration between the researchers and the subjects under study throughout the research process. The duration of the PAR process was four years (2003–2006). Childhood chronic disease was defined according to a three-axis framework [13], where the condition is assumed to have a biological, psychological or cognitive basis, being prolonged in time, and causing limitations in function or dependency on continuous therapy or services. The process involved researchers from medical informatics, paediatrics, and behavioural science, as well as clinicians and patients. Approximately ten two-hour design meetings were held each year, supported by separate small-group activities analysing specific design issues [14]. The core participatory design group consisted of practitioners, researchers, and engineers. Due to their limited availability, the contribution from children and parents to design process was constrained to sub-groups developing specific system functions or evaluating particular design questions. In addition to these physical meetings, the core participatory design group communicated with children and parents through interviews and mail surveys. At the initial stage in the design process, interviews, surveys, and cultural probe methods [15] were used to identify preliminary requirements and to explore alternative design elements. Individual design elements were transformed into system functions and associated with technical applications, e.g. weblogs [16]. In parallel, card sorting methods [17] were used to organize the design elements into more complex structures. The interconnected design patterns were represented textually in the form *Title, Context, Problem, Solution, Examples and References*. At the lowest level in each section of the pattern language, *Application references *were included in the graphical overview. When the overall system architecture was defined, sub-groups were formed to develop and formatively evaluate specific system functions, and the corresponding more particular design issues were progressively introduced into the pattern language. Finally, the system developers verified in iterative steps the inter-operability of system functions, and the evolving pattern language was refined by evaluations that also involved clinical staff, children, and parents. For the last representation of the revised pattern language, the final graphical overview was combined with corresponding tabulations of the textual design patterns. The design represented in the pattern language was progressively implemented in a Web 2.0 system during the development process. The resulting system  was introduced to patients, relatives, and caregivers and has been online since 2006.

## Results

The resulting design patterns describe a Web 2.0 architecture that supplies three main services to communities including children suffering from a chronic disease:

• access to resources for development and maintenance of the specific competences needed for management of the chronic disease in the community;

• endorsement of learning about the chronic disease management through online peer-to-peer communication;

• systematic accreditation of learning materials and processes

Two application-level preconditions were identified for implementation of the architecture in specific communities. First, the implementations have to be epidemiologically sensitive in order to support provision of age- and need-adjusted delivery of services on an equal basis to both resourceful and vulnerable groups of children. This means that the applications that constitute the interface between system content and the users must be chosen to suit the information infrastructure in the community and must be affordable for the broad majority of patients and their families. Second, to provide individualized support to children with a chronic disease, the system has to be accessible at all times and in all types of daily-life situations. Consequently, the applications cannot be restricted to only stationary computer platforms, but must also be available at different types of mobile devices, e.g. personal media players and cell phones.

**Figure 1:**
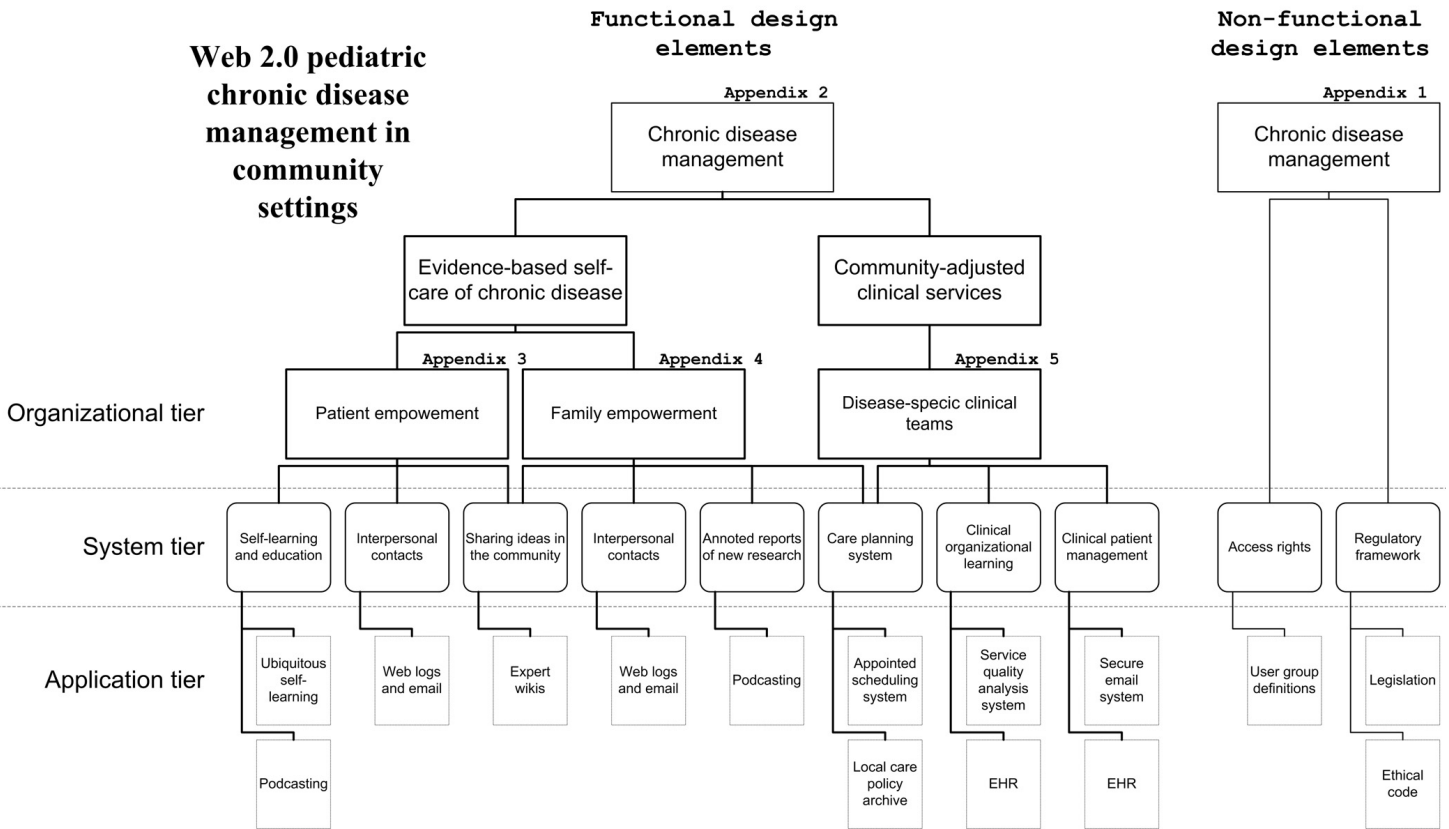
**Non-functional design elements are separated horizontally from the functional elements, while vertically the design patterns are divided into tiers for design elements at organizational, system, and application levels.** Appendices describing the design patterns are cross-referenced.

The design patterns used to define the system architecture were organized in an array structure (Figure 1). Vertically, non-functional design elements are separated from the functional elements, while horizontally the design patterns are divided into tiers for design elements at organizational, system, and application levels. The non-functional design elements mainly reflect the central information policy in the WHA eHealth resolution (Appendix 1). The *Access rights *design element highlights the fact that support of chronic disease management requires both ubiquitous access to the system for a broad category of users, and protection of the personal integrity of each user. In the *Regulatory framework *section, measures are described for harmonizing the Web 2.0 system with existing legislation and social norms.

The major part of the pattern language describes the functional design elements defining the Web 2.0 architecture. The design elements are at organizational level sub-divided into those associated with *Evidence-based self-care of chronic disease *and *Community-based clinical services *(Appendix 2). This division follows the social organization of daily practices in chronic disease management. Also following the way daily practice is structured, the design elements specific for evidence-based patient self-care are further categorized into those elements related to empowerment of patients and families, respectively (Appendix 3,4). Each organizational level element is thereafter specified by design elements at the systems and application levels. Correspondingly, the section of the Web 2.0 architecture outlining the organization of community-based clinical services to children with a chronic disease defines corresponding design elements at systems and application levels (Appendix 5).

## Discussion

The design patterns developed in this study show how practical, clinical, organizational, and technical knowledge about support of children with a chronic disease can be shared between patients, relatives, and caregivers using a Web 2.0 system compliant with the WHA eHealth resolution. The pattern language describes solutions to issues related to the design of Web 2.0 systems and visualizes some of the generic qualities of those solutions. Modern management of chronic disease, not the least Type 1 diabetes in children and adolescents, is today focused on the empowerment of individuals and groups in order to enable them to address the prevalent issues in daily life and their own local environment. A reliable and efficient information exchange between patients, parents, and clinicians is particularly important in this setting. The design patterns identified in this study mediate values that have been found to be important in community-based management of chronic disease, e.g. the value of allowing individuals to modify information services according to personal preferences and the value of experiencing 'belonging' to a larger community that shares one's own needs and interests [3].

Almost a decade ago, a shift in health care delivery from *biomedicine *to *infomedicine *was described as the *Country of PeoplePower*, an utopian scenario where communities, patients, and health workers joined in informed, shared governance over healthcare and clinical decision-making [18]. Drawing on ubiquitous computer communication technologies, patients and clinicians were foreseen to contribute actively to patient records, transcripts of clinical encounters were shared, and patient education occurred primarily in the home, in school and in community-based organizations. Patients and clinicians jointly developed 'quality contracts', serving as foundations for quality improvement systems that aggregate data, while also reflecting attributes of individual patients and clinicians. Exploiting Web 2.0 technologies and the present architecture, management of childhood chronic disease according to the *country of PeoplePower *scenario is no longer utopian. However, to realize such an *infomedical *shift in chronic disease care in local communities requires a focused effort involving a broad range of professional groups together with patients and their relatives. Even though central tenants of the Web 2.0 concept are self-evolution and networking, the core technical components must still respond to high software engineering standards. This implies that the implementation of the Web 2.0 architecture in local communities will require systematic approaches that allow capturing of broad perspectives on health service delivery and supporting multi-disciplinary decision-making, e.g. participatory design methods [14].

Systematic accreditation of learning materials is essential for a Web 2.0 system in the pediatric chronic disease setting. With the evolution of the Internet, traditional authorities have partly been replaced by *apomediaries*, which are tools and peers standing by to guide information seekers to trustworthy information, or adding credibility to information [19]. For apomediation to be a successful model for children with a chronic disease and their families, it has to be adjusted to the degree of maturity and autonomy of each child and parent. In Web 2.0 environments, the implementation of apomediary credibility at the application level may therefore become equal to or even more important than guaranteeing source credibility, e.g. as expressed by the HoN code [20]. This implies that there are several practical challenges for developers of pediatric chronic disease websites which aspire to come across as credible. Children and their parents need and want to be able to be co-creators of content, not merely an audience that is broadcasted to. Web 2.0 technology enables such sites. At the same time, to reduce late complications, modern clinical management of chronic disease is based on appropriateness and calibration principles [8,9], where the guidelines provided to patients are individualized and precise. Hence, developing credibility in the Web 2.0 context is also about developing the actual community in which the system is implemented. Chronic disease communities are built upon personal and social trust, and organized means to build and maintain that trust. It is thus necessary to find the appropriate balance in each community between patient co-determination and medical authority in chronic disease management, and to express this balance in the design of the apomediation components of the Web 2.0 system.

In the Web 2.0 context, accreditation issues are, however, not only limited to learning processes and content. Use of participatory design methods for implementation of Web 2.0 systems based on the architecture raises concerns about the management of responsibility in the total health service delivery perspective. Unlike system developers, practitioners and patients that participate in Web 2.0 system development and maintenance cannot be made professionally accountable for shortcomings in the system performance. In this context, it has to noted that the pattern language does not indicate which features require continuous organizational service offerings, and which can be managed by user communities, e.g. whether workspace assignments, addition of new software modules, and protection of existing software against hostile code can be administered by the user community. Already the original Internet's design relied on few mechanisms of central control. This lack of control has the added generative benefit of allowing new services to be introduced without up-front blocking by either individual users or public authorities [21]. To avoid downstream problems when implementing the pattern language in healthcare settings, ambiguities regarding the responsibility for software quality and maintenance need to be straightened out at an early stage. To mitigate the risk for late design failures, participatory design groups implementing Web 2.0 systems based on the pattern language have to include software engineering competences to cover areas such as system extensions and maintenance, and the design groups have to accept full responsibility for decisions made in these areas.

In design areas such as urban planning, pattern languages have been extensively used to transfer value-bearing features between different contexts [22]. A core set of design patterns constitutes the defining part of a pattern language developed for a particular area. Using the present language as a basis, a module-based Web 2.0 system design to support community-based childhood chronic disease management can be developed in different social and cultural contexts. Availability of high-level web programming languages, such as Joomla! , facilitates the software implementation process, providing access to modules corresponding to most components of the general architecture. The core set of design patterns delineate a smallest common denominator for the design specifications that are developed in order to implement particular systems. System developers in different community settings can then add the lower-level modules they need around this core in order to represent their actual program, and also add new sets of design patterns. In this way, each system design is tailor-made for its particular patient population and environment, while it still shares basic value-bearing features. Nevertheless, present high-level web programming languages are not adequate for development of all applications included in the pattern language. For instance, confidential patient-practitioner communication will in most settings require encryption to be added to the email messaging module. It is possible that over time, generic software modules can be developed for all application-level instances in the pattern language.

From a health informatics point of view, the most important feature of the pattern language is that it allows professional information system developers, clinical practitioners, and patients to share a common expression of the most important organizational and technical design issues during the development of Web 2.0 systems for use at the community level. What most previous methods, such as the Enterprise Architecture Framework [23] have in common is that they have been based on concepts developed for use by IS specialists. However, there is a substantial difference between an IS aimed to be used by professionals trained in formal analyses, and a Web 2.0 system that is to be shared by clinicians and laypersons of different ages at the community level. In the latter setting, identification of the needs of each different user group, lay and professional alike, is essential and requires that intelligible input can be delivered from all these users into the system development process. This, in turn, requires that the materials describing the system design are legible also for persons without a technical background.

The next step in the development of the Web 2.0 system design is the formative evaluation of the implemented prototype. This step is to address human-computer interaction and information content issues. A number of methods for evaluating health-related Web 2.0 systems are available, however, most of these use differing conceptual definitions and scoring approaches [24,25]. Additionally, the majority of methods proposed are also limited in their interpretability and ease of use, because they are usually defined to assess either health information content or usability from a human-computer interaction perspective. However, preliminary versions of comprehensive multi-disciplinary methods that cover both aspects have recently been published. For instance, the WebMedQual guideline for website assessment contains constructs for the assessment of information content, the authority of source, design, accessibility, links, user support, and privacy [26]. When such guidelines have been further developed into assessment standards, they will provide essential complements to pattern languages in the development of health and safety promotion websites.

## Conclusion

A pattern language representing a module-based architecture for Web 2.0 systems for paediatric chronic disease management has been described. Even though a high level of user participation in developing and managing content is the core of the Web 2.0 concept, the WHA eHealth resolution requires that this participation is structured and that the medical quality of the services provided is assured. The use of design patterns allows representing the core design elements of a Web 2.0 system upon which an 'ecological' development of content respecting these constraints can be built. Future research should include evaluations of Web 2.0 systems implemented according to the architecture in practice settings.

## Competing interests

The authors declare that they have no competing interests.

## Authors' contributions

TT conceived of the study, led the system design, and drafted the first version of the manuscript. HE led the implementation of the system. JL led the clinical application of the system. JE managed the graphical language during the system design. SN was responsible for the clinical content of the system. LH managed the interaction with practitioners and patients during the system design. All authors contributed to later drafts and approved the final manuscript.

## Appendices

### Appendix 1. Non-functional design elements

#### Access rights

Context: Chronic disease management.

Problem: Everyone with an interest in the management of a specific chronic disease should have access to the Web 2.0 system. However, patients and their parents are mainly interested in practical advice and education, while clinicians need access to confidential patient data and information in their daily work.

Solution: The Web2.0 system is to be divided into virtual workspaces, where each individual entering the system is assigned a collaborative workspace that contains functions related to user resources, interests, and qualifications in chronic disease management and computer use.

Examples: In the diabetes care setting, each user, lay and professional alike, is provided with a username and a password to be able to access personal sections of the system. Each time the user logs on to the system, resources adapted to their interests are displayed.

Application reference: User group definitions.

#### Regulatory framework

Context: Chronic disease management

Problem: Chronic disease management may threaten integrity and privacy of individuals and organizations. It is a basic human right that individual health data are treated confidentially and not shared without consent.

Solution: In order to assess threats to personal integrity in pervasive health surveillance, an advisory group on ethics and legal issues is to be formed in association with the Web 2.0 system. The group should represent the general public, medicine, ethics, IT security and non-medically trained public health professionals.

Examples: In the paediatric diabetes setting, one person is responsible for decisions regarding the establishment of personal index files and inquiries about data from these files. The intention is to form a permanent multi-disciplinary advisory panel on ethical and IT security issues.

Application references: Ethical code, Legislation.

### Appendix 2. Functional organizational level design elements

#### Chronic disease management

Context: Chronic disease is a significant problem both for the affected children and their families and healthcare providers.

Problem: Even though there is scientific evidence that certain clinical and self-management strategies are effective in specific disease categories, this knowledge has not been used to change practice. There is a general lack of knowledge at local, national and international levels about which interventions are effective in which settings. Additionally, it is hard for both patients and clinical professionals to learn how peers have solved common problems.

Solution: A Web 2.0 system that is to provide patients and their caretakers with continuous access to a chronic disease management program that provides both support for self-management of the chronic disease in daily life, and access to just-in-time clinical services.

Examples: In the diabetes care setting, the Web 2.0 system is divided into a website that supports self-management, and a patient-centred health information system (HIS) adapted to support community-based clinical services.

References: Evidence-based self-management of chronic disease, Community-based clinical services.

#### Evidence-based self-management of chronic disease

Context: Chronic disease management.

Problem: Self-management of chronic disease often suffers from insufficient integration and influences from invalidated knowledge.

Solution: The Web 2.0 system is to provide virtual collaborative workspaces for the empowerment of those interested in specific areas of the self-management of chronic disease. These workspaces will provide tools for communication and collaboration, learning, searching for information, and an archive of peer-reviewed publications.

Examples: The workspaces in the diabetes care setting are to be accessible to different groups of patients, family members, and others in the patients' social environment. The workspaces are sub-divided between children and adults, as well as according to geographic region.

References: Patient empowerment, Family empowerment.

#### Community-based clinical services

Context: Chronic disease management

Problem: Most communities have insufficient knowledge of age-specific prevalence of specific chronic diseases. This lack of knowledge is an obstacle to the implementation of high-quality clinical services adapted to the needs in particular communities, i.e. that can support all children in all life situations.

Solution: A four-stage process is to be used to organize community-based chronic disease management, utilizing epidemiological surveys at the community level as a foundation for resource planning. The first stage is to concern establishing a baseline and deciding priorities, while the second stage will involve setting up an organizational structure, defining health goals, and designing interventions targeted at the identified goals. The third stage is to establish a time-line and provide care resources. Finally, the interventions are to be evaluated and results disseminated.

Examples: The management planning of diabetes Type 1 in Östergötland. Sweden, is county-based and provided by multi-professional teams. Each patient and family is associated with the geographically closest team.

References: Disease specific clinical teams

### Appendix 3. Design elements addressing the empowerment of children with a chronic disease

#### Patient empowerment

Context: Evidence-based self-management of chronic disease.

Problem: A child with a chronic disease can benefit from interaction with many persons and resources distributed over many different settings and organizations in a community. This diversity causes difficulties in optimally utilizing existing resources and competencies.

Solution: Tools for interactive learning and structured communication that enhance the establishment of formal and informal social networks are to be included in the Web 2.0 system. In addition, wikis and weblogs will provide the means to create virtual communities for knowledge exchange.

Examples: In the diabetes type 1 context, the Web 2.0 system mainly supports the user's personal learning using validated materials, but it also provides support for communication with peers and the local diabetes team. The system is in part implemented using the Open Source Content Management Systems Joomla.

References: Sharing ideas in the community, Interpersonal contacts, Ubiquitous self-learning.

#### Interpersonal contacts

Context: *Patient empowerment*

Problem: Children with a chronic disease may have a lower self-esteem and self-efficacy than their healthy peers.

Solution: Children will be able to create their own personal weblogs in the Web 2.0 system. Personal e-mail will be supported at several places in the system to make it possible to communicate also with clinical practitioners in without entering into any of the delimited workspaces.

Examples: In the diabetes type 1 context, each child is given the possibility to express her/himself to the local community of diabetic peers through a personal webpage. The e-mail feature is implemented into traditional e-mail applications.

Application references: Weblogs (blogs), email.

#### Sharing ideas in the community

Context: *Patient empowerment*

Problem: There is a lack of locally adjusted expert information regarding living with a chronic disease.

Solution: Expert wikis are to be provided as a semi-formal way of informing others in the community. This function is to constitute a complement to more general podcasts and interactive learning materials.

Examples: In the diabetes Type 1 context, several types of *wikis *are available. The understanding is that short notes contributed by experts in the community, rather than long reports, are often sufficient to keep other children informed about new ideas, experiences, and ongoing activities.

Application references: Wikis.

#### Ubiquitous self-learning

Context: *Patient empowerment*

Problem: Children with a chronic disease are usually taught about their condition by their parents, directly by their clinical caretakers, from documents, e.g. pamphlets, reports, and science-based newspaper articles. Children seldom find these printed materials interesting, and the materials are sometimes not even distributed to those who might find them useful.

Solution: Podcasting of electronic learning materials is to be combined with a filing system for organizing the learning resources, a search function, and the potential to browse content. These resources could be downloaded to different types of personal media players, cell phones, and computers.

Examples: An interactive diabetes simulation application is available for learning about how different insulin types interact with food intake and physical activity. Digitalized video materials are available for downloading.

Application references: Interactive learning systems, Podcasting.

### Appendix 4. Design elements that address support to families with a child with a chronic disease

#### Family empowerment

Context: Evidence-based self-management of chronic disease.

Problem: The importance of relevant and accessible information about services to families with disabled children is well established. Keeping families informed is a significant factor both for empowerment and for enabling participatory service processes. Nevertheless, even though there has been considerable research that highlights parents' information needs, there has been less exploration of how parents would actually like to receive this information.

Solution Tools for interactive learning and structured communication that enhance the establishment of formal and informal social networks are to be included in the Web 2.0 system. Additionally, wikis and weblogs will provide the means to create virtual communities for knowledge exchange.

Examples: In the diabetes Type 1 context, the IS mainly supports the user's personal learning, but it also provides support for communication with peers and the local diabetes team.

References: Interpersonal contacts, Annotated reports of new research, Care planning system.

#### Interpersonal contacts

Context: *Family empowerment*

Problem: Parents with children with a chronic disease often have difficulties getting in touch with peers to share experiences.

Solution: Parents can be able to create their own personal weblog in the Web 2.0 system. Personal e-mail is to be supported at several places in the system to make it possible to communicate also with clinical practitioners without entering into any of the delimited workspaces.

Examples: In the diabetes Type I support system, parents can choose to commit themselves to sharing experiences with other parents and other adults involved with chronically ill children. Several communication tools are available for sharing experiences.

Application references: Weblogs (blogs), email.

#### Annotated reports of new research

Context: *Family empowerment*

Problem: It is difficult for parents to obtain an overview of new research findings on the chronic disease, the quality of these findings, and how they can be accessed and assessed.

Solution: A clearinghouse function in the Web 2.0 system is to represent a news archive and a repository of downloadable materials. New materials are to be collected and annotated by a qualified researcher. The materials can either be read online or downloaded automatically or upon demand.

Examples: In the diabetes Type I support system, the research news management is divided into specific areas such as basic science, pharmacology, and biomedical engineering. The information can be stored in different formats in the clearinghouse as, e.g. text, graphs, tables, and multi-media clips.

Application references: Podcasting.

#### Care planning system

Context: *Family empowerment*

Problem: Clinicians must understand patient priorities to create an effective treatment partnership. Physicians have been found to underestimate the importance adolescents place on communicating with their physician as a friend and medical-technical aspects of care.

Solution: The Web 2.0 system is to be used both to inform families about the current organization of care provision and to survey families for their experiences and preferences.

Examples: In Östergötland, the patients and their families are regularly surveyed for their care experiences and preferences. Care policy documents are available for reading or downloading

Application references: Appointment scheduling system, Local care policy archive.

### Appendix 5. Design elements that address support to disease-specific clinical teams

#### Disease-specific clinical teams

Context: Community-based clinical services

Problem: Management of children with a chronic disease poses complex clinical problems that can seldom be used within the context of one profession only.

Solution: Interprofessional healthcare is expected to generate health gains through better responsiveness to patients, more efficient use of resources, and the enhancement of patient safety. Community-based clinical services provided to children with a chronic disease are associated primarily with working in teams. The task of individual team members is thus also to reform healthcare routines where conventionally organized care has failed. Team members are expected to manage among themselves the competing political agendas upon which their various professional identities are based. In parallel, they are expected to manage the team processes that occur in all teamwork.

Examples: The diabetes teams in Östergötland consist of paediatricians, specialist nurses, and dieticians. There are 3 teams distributed at three hospitals, caring for 600 patients.

References: Clinical patient management, Clinical quality assurance.

#### Clinical patient management

Context: *Disease-specific clinical teams*

Problem: The clinical management of childhood chronic disease requires medical, behavioural, and social competences, and thus cooperation between different professional categories. These multi-professional teams need to share a common care documentation system.

Solution: An Electronic Health Record (EHR) is to be introduced that can be accessed and used by the different professionals in the community involved in the care of children with a chronic disease. Patients with chronic disease are to be provided with web-based access to their own records. Patients will be able to leave messages to their clinical teams through a secure email system.

Examples: In Östergötland, an integrated EHR is accessible from all clinics throughout the county. Patients with chronic disease are provided specific and secure access to their own records from home. A web portal supports messaging to specific chronic disease teams regarding appointment times and other administrative issues.

Application references: Integrated EHR, Secure email system.

#### Clinical organizational learning

Context: *Disease-specific clinical teams*

Problem: There is insufficient potential to analyze differences in clinical outcomes with regard to geographic area and socioeconomic background for children with a chronic disease.

Solution: An EHR is to be extended with a service quality analysis module to allow definition of quality indicators and analyze data. This functionality will enable clinical teams to develop their clinical service provision based on their own resources and organizational conditions.

Examples: In the diabetes Type I setting in Östergötland, the quality of the clinical care is monitored using time-series analyses of the indicator biomedical variables, e.g. daily B-glucose curves and weekly progressions of HbA1c. Both service quality and the general quality of life of patients are surveyed bi-annually using validated instruments, such as EuroQol.

Application references: Integrated EHR, Service quality analysis system.

## Pre-publication history

The pre-publication history for this paper can be accessed here:



## References

[B1] Sawyer SM, Drew S, Yeo MS, Britto MT (2007). Adolescents with a chronic condition: challenges living, challenges treating. Lancet.

[B2] Barlow JH, Ellard DR (2004). Psycho-educational interventions for children with chronic disease, parents and siblings: an overview of the research evidence base. Child Care Health Dev.

[B3] Castells M (1998). End of millennium.

[B4] Timpka T (2001). Proactive health computing. Artif Intell Med.

[B5] Bång M, Larsson A, Berglund E, Eriksson H (2005). Distributed user interfaces for clinical ubiquitous computing applications. Int J Med Inform.

[B6] Boulus N, Bjorn P (2007). Constructing technology-in-use practices: EPR-adaptation in Canada and Norway. Stud Health Technol Inform.

[B7] McLean R, Richards BH, Wardman JI (2007). The effect of Web 2.0 on the future of medical practice and education: Darwikinian evolution or folksonomic revolution?. Med J Aust.

[B8] Bojestig M, Arnqvist HJ, Hermansson G, Karlberg BE, Ludvigsson J (1994). Declining incidence of nephropathy in insulin-dependent diabetes mellitus. N Engl J Med.

[B9] Loveman E, Cave C, Green C, Royle P, Dunn N, Waugh N (2003). The clinical and cost-effectiveness of patient education models for diabetes: a systematic review and economic evaluation. Health Technol Assess.

[B10] Gamma E, Helm R, Johnson R, VJ (1995). Design patterns: elements of reusable object-oriented software.

[B11] Buschmann F, Meinier R, Rohnert H, Sommerlad P, Stal M (1996). A System of Patterns: Pattern-Oriented Software Architecture.

[B12] Foothe-Whyte W (1991). Participatory Action Research.

[B13] Stein RE, Bauman LJ, Westbrook LE, Coupey SM, Ireys HT (1993). Framework for identifying children who have chronic conditions: the case for a new definition. J Pediatr.

[B14] Pilemalm S, Timpka T (2008). Third generation participatory design in health informatics – making user participation applicable to large-scale information system projects. J Biomed Inform.

[B15] Hassling L, Nordfeldt S, Eriksson H, Timpka T (2005). Use of cultural probes for representation of chronic disease experience: exploration of an innovative method for design of supportive technologies. Technol Health Care.

[B16] Graspemo G, Hassling L, Nordfeldt S, Eriksson H, Timpka T (2004). Design of interactive health drama built on social realism. Stud Health Technol Inform.

[B17] Rugg G, McGeorge P (1997). The sorting techniques: a tutorial paper on card sorts, picture sorts and item sorts. Expert Systems.

[B18] Delbanco T, Berwick DM, Boufford JI, Edgman-Levitan S, Ollenschlager G, Plamping D, Rockefeller RG (2001). Healthcare in a land called PeoplePower: nothing about me without me. Health Expect.

[B19] Eysenbach G (2007). From intermediation to disintermediation and apomediation: new models for consumers to access and assess the credibility of health information in the age of Web2.0. Stud Health Technol Inform.

[B20] Boyer C, Geissbuhler A (2005). A decade devoted to improving online health information quality. Stud Health Technol Inform.

[B21] Zittrain J (2008). The future of the Internet and how to stop it.

[B22] Alexander C (1979). The timeless way of building.

[B23] O'Carroll PW, Cahn MA, Auston I, Selden CR (1998). Information needs in public health and health policy: results of recent studies. J Urban Health.

[B24] Silberg WM, Lundberg GD, Musacchio RA (1997). Assessing, controlling, and assuring the quality of medical information on the Internet: Caveant lector et viewor – Let the reader and viewer beware. Jama.

[B25] Eysenbach G, Powell J, Kuss O, Sa ER (2002). Empirical studies assessing the quality of health information for consumers on the world wide web: a systematic review. Jama.

[B26] Provost M, Koompalum D, Dong D, Martin BC (2006). The initial development of the WebMedQual scale: domain assessment of the construct of quality of health web sites. Int J Med Inform.

